# Hepatic Transcriptome Variations Among Different Evolutionary Lineages of *Rhinolophus ferrumequinum* During Hibernation

**DOI:** 10.3390/biology15050425

**Published:** 2026-03-05

**Authors:** Yue Zhu, Sen Liu, Jianying Du, Yanhong Xiao, Keping Sun

**Affiliations:** 1 Jilin Provincial Key Laboratory of Animal Resource Conservation and Utilization, Northeast Normal University, Changchun 130117, China; zhuy309@nenu.edu.cn (Y.Z.);; 2 College of Life Sciences, Henan Normal University, Xinxiang 453007, China; 3 Jilin Provincial International Cooperation Key Laboratory for Biological Control of Agricultural Pests, Changchun 130118, China

**Keywords:** greater horseshoe bat, hibernation, hepatic transcriptome, evolutionary lineage

## Abstract

The greater horseshoe bat (*Rhinolophus ferrumequinum*), which is widely distributed across the temperate regions of China, primarily consists of two major evolutionary lineages: a northeastern (NE) lineage with a hibernation period of 6–8 months and a central–eastern (CE) lineage with a hibernation period of 4–5 months. This study conducted a comparative analysis of liver transcriptomes from these two lineages during the active, torpor, and arousal phases. The results indicated that the CE lineage exhibited a significantly greater number of differentially expressed genes (DEGs) compared to the NE lineage. During the torpor phase, both lineages transitioned from carbohydrate metabolism to lipid metabolism, substantially downregulating genes and pathways associated with amino acid metabolism, and upregulating immune-related genes to maintain essential defense functions. In the arousal phase, both lineages only moderately activated several genes associated with immunity and metabolic regulation to facilitate a rapid return to torpor. Notably, the number of DEGs co-regulated between the two lineages was very limited, and a large number of lineage-specific regulatory genes related to energy and metabolism were identified. This may reflect the adaptability of different bat lineages to the local environment, highlighting the importance of habitat conditions in lineage differentiation. Therefore, hibernation induces substantial transcriptomic reorganization in the liver of *R. ferrumequinum*, particularly affecting metabolic and immune processes. Distinct geographic lineages exhibit unique hibernation adaptation strategies through the regulation of specific genes and pathways. This study enhances the understanding of the molecular mechanisms underlying hibernation adaptation across different evolutionary lineages of the same species at the transcriptomic level, providing insights into the evolutionary adaptations of animals to environmental changes.

## Simple Summary

The northeastern and central–eastern lineages of *Rhinolophus ferrumequinum* exhibit significantly divergent sets of hepatic differentially expressed genes and pathways, with minimal overlap during the torpor and arousal phases. This indicates lineage-specific transcriptomic adaptations to their respective climatic conditions and varying durations of hibernation.

## 1. Introduction

The greater horseshoe bat (*Rhinolophus ferrumequinum*) is a species of Chiroptera widely distributed across mainland China. During its evolutionary history, this species initially expanded into the southwestern region of China, gradually moving northward across the Qinling Mountains, and eventually reaching the northeastern part of the country [1]. Geographical barriers such as the Qinling Mountains and the eastern sea have led to the emergence of three distinct and independent evolutionary lineages: the southwestern (SW), central–eastern (CE), and northeastern (NE) lineages [1,2,3]. Notable adaptive divergence has been observed among them: the SW lineage remains active year-round, whereas both the CE and NE lineages exhibit hibernation behavior.

Previous transcriptomic studies on hibernation in *R. ferrumequinum* have largely focused on specific populations and have revealed tissue-specific gene expression patterns during hibernation. For instance, in the brain, genes associated with glycolysis, electron transport, protein folding, oxidative stress, and cytoskeleton organization differ between active and hibernating states [4]. In the liver, genes related to metabolic suppression, immune function, and stress response are affected [5]. Sun et al. [6] extended their research to examine intestinal transcriptomes across four distinct physiological states: summer active, torpor, arousal, and winter active. Their findings indicate that hibernation is generally characterized by the downregulation of carbohydrate metabolism, the upregulation of lipid metabolism, and the enhancement of immune-related genes to resist pathogenic threats. Conversely, during the arousal phase, there is an upregulation of genes associated with thermoregulation, immunity, and oxidative stress, which facilitates a swift physiological transition.

However, these findings are primarily based on a single lineage (e.g., the CE lineage), making it unclear whether the expression pattern is consistent across different evolutionary lineages of *R. ferrumequinum.* Notably, compared to the CE lineage, the NE lineage has adapted to colder climates and consequently evolved a longer hibernation period (approximately 6–8 months for the NE lineage and 4–5 months for CE lineage). Although there is currently no consensus on the differences in hibernation strategy phenotypes between the NE and CE lineages, existing research suggests potential genetic and physiological differences [7]. Such lineage-specific adaptations, resulting from long-term evolutionary isolation and environmental differences, indicate potential divergence in the molecular regulation of hibernation.

Therefore, this study aimed to address a significant and underexplored question: Do the magnitudes of liver transcriptomic changes during hibernation and arousal differ significantly between distinct evolutionary lineages—the CE and NE lineages? We hypothesized that long-term evolutionary isolation and environmental variation have led to (1) significant inter-lineage differences in gene expression that are adaptive to specific hibernation behaviors, and (2) more extensive gene silencing in the northeastern lineage to support its extended hibernation duration. To test these hypotheses, we performed a comparative transcriptome analysis on *R. ferrumequinum* from the CE and NE lineages across three distinct physiological stages: active, torpid, and arousal. This research provides molecular insights into how different lineages adapt to their specific hibernation behaviors and physiological stresses through transcriptional regulation, offers new perspectives for understanding the evolution of hibernation behavior, and supplies foundational data for related studies on hypoxia, cold tolerance, and ischemia–reperfusion injury.

## 2. Materials and Methods

### 2.1. Sample Collection

Samples from the CE lineage were collected from a cave in the Henan Taihangshan Macaque National Nature Reserve in Jiyuan City, Henan Province, China. The annual average temperature is 14.8 °C, and the humidity is 98.8%. Samples from the NE lineage were collected from Temple Cave, Huanren County, Liaoning Province, China. The annual average temperature is 9.4 °C and the humidity is 98.3%. The active groups, sampled in August, consisted of bats in a homeothermic state. Both the hibernation (torpid) and arousal groups were sampled in January. During this period, torpid bats were in a heterothermic state with body temperatures approximating ambient conditions, whereas arousal bats had reverted to a normothermic state post-awakening. Within the CE lineage, the groups were designated as HN (active), HH (torpid), and HHW (arousal), representing populations from Henan. Similarly, within the NE lineage, the groups were labeled as LN (active), LH (torpid), and LHW (arousal), representing populations from Liaoning Province. To avoid potential group effects, we only collected adult *R. ferrumequinum* from both lineages, including both male and female individuals at each physiological stage (Table S1). All individuals were euthanized directly at the sampling site, weighed, and had their forearm lengths measured. Liver tissues were quickly extracted, placed in cryogenic tubes, preserved in liquid nitrogen, transported to the laboratory, and stored in a −80 °C freezer. The sample names consist of letters and numbers. Due to contamination of one individual’s tissue in the LH group, it was excluded from sequencing and analysis.

### 2.2. Total RNA Extraction and High-Throughput Sequencing Library Construction

Approximately 0.02 g of liver tissue was taken from each sample, and total RNA was extracted using the Efficient Animal Tissue Total RNA Extraction Kit (Beijing Genesand Biotech Co.,Ltd,. RE715, Beijing, China). The effectiveness of RNA extraction was assessed via electrophoresis on a 1% agarose gel, and concentrations were measured using a Nanodrop. After passing the quality inspection, library construction was performed, followed by preliminary quantification using a Qubit 2.0 Fluorometer (Thermo Fisher Scientific Corporation, Waltham, MA, USA). The library was then diluted to 1.5 ng/μL for Illumina sequencing.

Raw data were filtered to remove reads containing adapters, reads with ambiguous base calls (denoted as N), and low-quality reads (where bases with a Phred quality score ≤ 20 account for more than 50% of the read length). Quantitative analysis was performed using the featureCounts tool (v1.5.0-p3) from the Subread software package.

A total of 35 RNA extractions and reference transcriptome sequencing were completed, generating 232.47 Gb of data after quality control of the raw data. The clean data from each sample exceeded 6 Gb, with Q30 base percentages greater than 93.29% and GC content around 50%. The overall alignment rate ranged from 88% to 91.32% (Table S2). Gene expression quantification was expressed as normalized counts, which grouped replicate samples together more accurately than TPM and FPKM [8].

### 2.3. Statistical Analyses

Principal Component Analysis (PCA) was performed on the normalized counts table to visualize overall variation between groups. Differential gene expression analysis between specific groups of the two lineages was conducted using DESeq2 package in R software (v4.2.3). The criteria for screening differentially expressed genes (DEGs) were |log2FoldChange| > 1 and *p*-adj (False Discovery Rate) < 0.05. The transcriptome differential analysis included four comparisons: HN vs. HH and HH vs. HHW in the CE lineage, and LN vs. LH and LH vs. LHW in the NE lineage, followed by clustering the DEGs. A protein–protein interaction (PPI) network of the DEGs associated with physiological stages was constructed using the STRING database (https://STRING-db.org/). The top 10 ranked genes in the PPI network were selected as key genes using the MCC algorithm from the CytoHubba plugin in Cytoscape (v3.10.4).

Co-regulated DEGs between the same treatment groups of the two lineages were then used to identify core genes involved in hibernation in *R. ferrumequinum*. Furthermore, integrating two lineages, three physiological stages, and their interactions, we constructed a DESeq2 time course model to identify genes exhibiting lineage-specific regulation. Based on the regulatory patterns, a heatmap will be generated for this set of genes, along with functional enrichment analysis. Functional enrichment analysis was then performed on these genes based on their regulatory patterns. Gene enrichment analysis using GO (Gene Ontology) and KEGG (Kyoto Encyclopedia of Genes and Genomes) was performed with clusterProfiler software (v3.4.4), setting *p*-adj < 0.05 as the criterion for significance. 

## 3. Results

### 3.1. Principal Component Analysis

**Figure 1:**
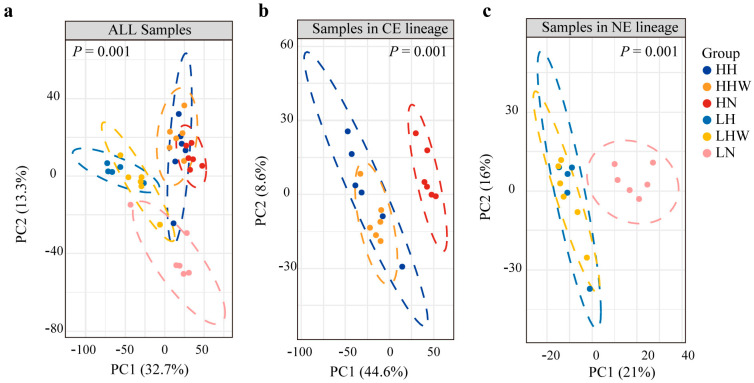
Analysis of liver gene expression patterns of *R. ferrumequinum* in different lineages across distinct physiological stages. Principal Component Analysis (PCA) in all samples (**a**), samples from the CE lineage (**b**), and samples from the NE lineage (**c**).

PCA effectively distinguished individuals from two lineages, with the first two components accounting for 47.09% of the total variance (Figure 1a).

In the CE lineage, active individuals were clearly separated from those in torpid and arousal states, which were not distinctly different. Conversely, individuals in the NE lineage were more clustered, showing less dispersion than the CE lineage (Figure 1a). This difference may be due to the varying number of DEGs between the groups. A separate PCA for each lineage showed similar patterns: active individuals were distinct from torpid and arousal groups, which overlapped significantly (Figure 1b,c). Despite physiological normalization, the gene of expression of arousal group remained similar to that of the torpid group, aiding rapid hibernation resumption in *R. ferrumequinum*. ANOSIM analysis supported the PCA results, with a significance level of *p* values < 0.01.

### 3.2. Differentially Expressed Genes

**Figure 2:**
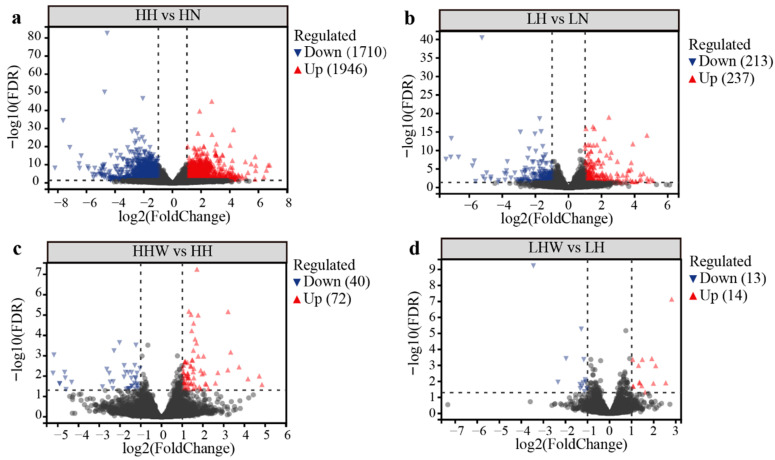
DEGs between different physiological stages. DEGs in bats from the CE and NE lineages (**a**,**b**) that enter hibernation from active phase, and DEGs in bats from the CE and NE lineages (**c**,**d**) that arousal from torpor. Red indicates upregulation, blue indicates downregulation, and gray indicates genes with no significant differences.

In comparing torpid to active states, 3656 DEGs were identified in the CE lineage (HH vs. HN), with 1946 genes upregulated and 1710 genes downregulated in torpid individuals (Figure 2a, Figure S1). The upregulated genes are predominantly enriched in coagulation and fibrinolysis regulation (e.g., *FGA*, *FGB*, *FGG*), immune functions (e.g., *Hp*, *A2m*) and oxidative stress response (e.g., *GPX3*, *CP*). Conversely, the downregulated genes are primarily associated with metabolic processes (e.g., *MAT1A*), iron metabolism (e.g., *FTH1*, *HPX*) and lipid transport (e.g., *PCK1*, *APOE*) (Table S3). In contrast, the NE lineage exhibited 450 DEGs, consisting of 237 upregulated and 213 downregulated genes in torpid individuals (LH vs. LN, Figure 2b, Figure S2). The upregulated genes in the NE lineage are mainly related to metabolism (e.g., *GLUL*), and immunity (e.g., *HPGD*, *SDS*, *HAMP*, *LEAP2*), while the downregulated genes are associated with amino acid catabolism (e.g., *TAT*, *PAH*, *GOT1*, *OAT*), heme binding (e.g., *HAMP*) and gluconeogenesis (e.g., *G6pc*) (Table S4).

In the comparison between arousal and torpid states, a total of 112 DEGs were identified in the CE lineage, with 72 genes being upregulated and 40 genes downregulated in the arousal individuals (Figure 2c, Figure S3). The upregulated genes predominantly pertain to energy metabolism (*PDP2*), oxidative stress response (*NFE2L2*), and immune functions (e.g., *HSPH1*, *NOD2*, *HMOX1*). In contrast, the downregulated genes are largely linked to stress response and signal transduction (*GADD45B/G*, *MAP3K8*) and regulation of the cell cycle and proliferation (*MN1*), as well as transcription and rhythmic regulation (*BHLHE40*) (Table S5). Within the NE lineage, 27 DEGs were identified, with 14 genes upregulated and 13 genes downregulated in the arousal individuals (Figure 2d, Figure S4). The upregulated genes are primarily involved in cell proliferation (*CDK1*, *NEK10*), immune (*PDE4B*) and metabolic regulation (*MCC*, *NNAT*). The downregulated genes are associated with the suppression of metabolism-related gene expression (*FGF21*) and zinc finger transcription factor (*ZNF274*, *ZFP3*) (Table S6). Cluster analysis conducted on the basis of DEGs reveals that samples from the same lineage are grouped according to their physiological stages (Figures S1–S4).

PPI analysis shows that key genes are related to physiological stages and lineage. During the transition from active state to torpor, the key genes in the CE lineage are all core regulatory factors involved in cell division, with most genes exhibiting low expression. In contrast, the key genes in the NE lineage are overexpressed and primarily associated with interferon-stimulated genes (ISGs), including *ISG15*, *OASL*, and *MX2*. After arousal from torpor, the key genes in the CE lineage are involved in the cellular adaptive response to environmental stimuli, with most genes being overexpressed, including *TRIB1* and *NFE2L2*. The DEGs network in the NE lineage has fewer than two network edges, and was thus excluded from further analysis (Table S7).

Notably, 107 upregulated and 79 downregulated DEGs are co-regulated between the two lineages in torpid individuals, potentially representing core genes that mediate the torpid state in *R. ferrumequinum*. Certain immune-related genes (*S100A8/A9*, *BIRC3*, *TRIM14*) and stress response genes (*AOX1*) are consistently upregulated (Table S3). Conversely, DEGs associated with gluconeogenesis (*G6PC*), metabolism (*TAT*, *GOT1*), immunity (*CCL16*, *CD74*), and development (*GADD45G*, *IGFBP2*) exhibit downregulation (Table S8). The clustering heatmap of co-regulated DEGs reveals that, compared to lineage differences, samples are preferentially clustered based on the same physiological state (Figure S5). During the torpor–arousal process, no DEGs were found to be co-regulated in both lineages. Furthermore, we identified a total of 3813 genes exhibiting lineage-specific regulation, categorized into four regulatory modes (Figures S6 and S7). Compared to the CE lineage, 1475 genes in the NE lineage showed consistently enhanced responses, while 1494 genes exhibited persistently weakened responses. Additionally, 844 genes demonstrated differential responses that were specific to certain developmental stages (Table S9).

### 3.3. GO Enriched Terms

**Figure 3:**
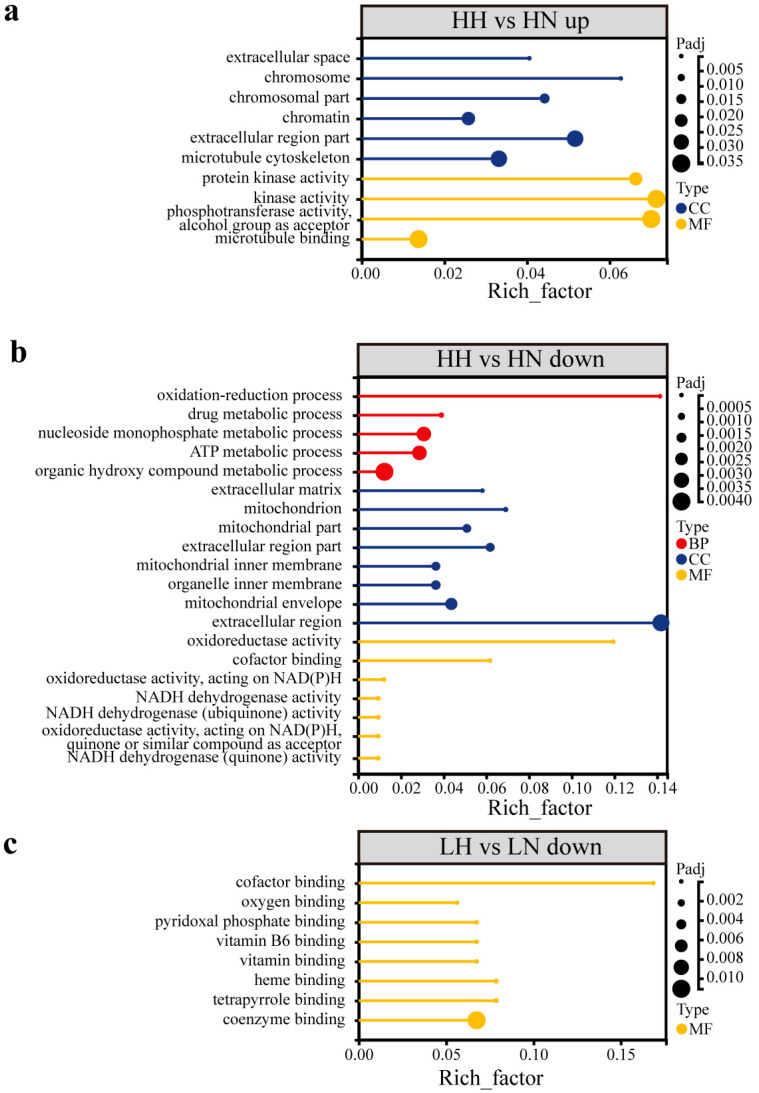
GO enrichment analysis in torpid vs. active states. GO terms for upregulation (**a**) and downregulation (**b**) in HH; the downregulation GO terms in LH (**c**).

**Figure 4:**
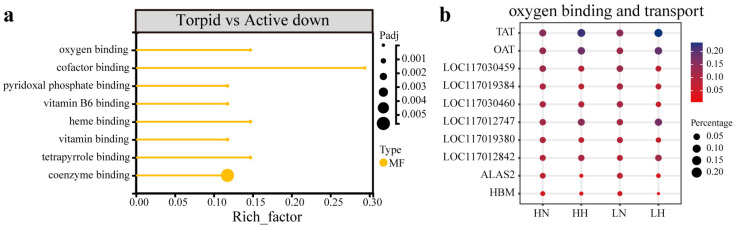
Functional enrichment analysis of DEGs co-regulated in the CE and NE lineages. GO enrichment analysis based on co-regulated DEGs in torpid vs. active states (**a**), and a bubble plot of normalized counts of genes in each group (**b**).

In the comparison between torpid and active states, the CE lineage has ten upregulated significantly enriched terms, primarily associated with the extracellular space (GO: 0005615, 0044421), the cytoskeleton (GO: 0015630) and binding (GO: 0008017) (Figure 3a). Concurrently, there are 70 downregulated GO terms, primarily associated with metabolites, biosynthesis, and transmembrane transport (Figure 3b, Table S10). In contrast, the NE lineage has only eight significantly downregulated enriched terms (Table S11), related to binding for coenzymes (GO: 0048037, 0050662), vitamins (GO: 0030170, 0070279, 0019842), and heme (GO: 0019825, 0020037, 0046906) (Figure 3c). Among the co-regulated DEGs, all eight significantly downregulated entries are related to oxygen binding and transport (Figure 4, Table S12). This finding suggests that bats from diverse lineages may commonly adopt a strategy of downregulating oxygen-related metabolic processes during hibernation to adapt to hypoxic conditions.

**Figure 5:**
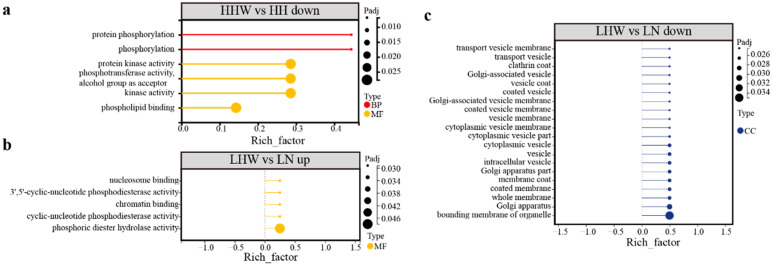
GO enrichment analysis in arousal vs. torpid states. The downregulation GO terms in HHW (**a**); GO terms for upregulation (**b**) and downregulation (**c**) in LHW.

In the comparison between arousal and torpid states, there are only six significantly downregulated terms in the CE lineage (Figure 5a), which are related to protein phosphorylation, protein kinase activity, and phospholipid binding (Table S13). In contrast, the LHW versus LH comparison revealed five enriched upregulated terms, including those related to phosphodiesterase activity (primarily involving *PDE4B*) and nucleosome and chromatin binding (primarily involving *SMARCA1*) (Figure 5b). Meanwhile, 25 downregulated terms were enriched in the LHW vs. LH comparison (Figure 5c), predominantly encompassing membrane and vesicle coat functions, largely driven by significant enrichment associated with the gene *CLTCL1* (Table S14).

The Both_Pos gene module enriched 126 significantly altered terms, primarily involving the oxidation–reduction process, ATP metabolic process, and carbohydrate metabolic process; the Both_Neg gene module enriched 43 significantly altered terms, mainly related to protein localization, intracellular transport, and GTP binding; the HvsN_Only_Pos gene module was primarily associated with 14 significantly altered terms related to ribonucleoprotein complex biogenesis, ncRNA metabolic process, and methyltransferase activity. The WvsH_Only_Pos enriched seven significantly altered terms, mainly related to phospholipid binding and protein kinase activity (Table S15).

### 3.4. KEGG Enriched Pathways

**Figure 6:**
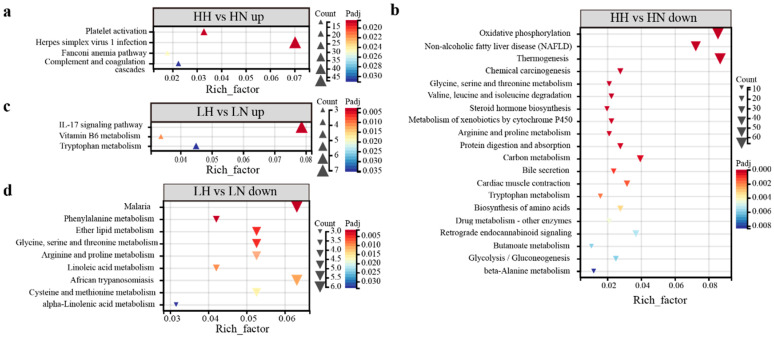
KEGG enrichment pathways of DEGs in torpid vs. active states. Upregulation (**a**) and downregulation (**b**) pathways in CE lineage bats; The pathways upregulated (**c**) and downregulated (**d**) in NE lineage bats.

**Figure 7:**
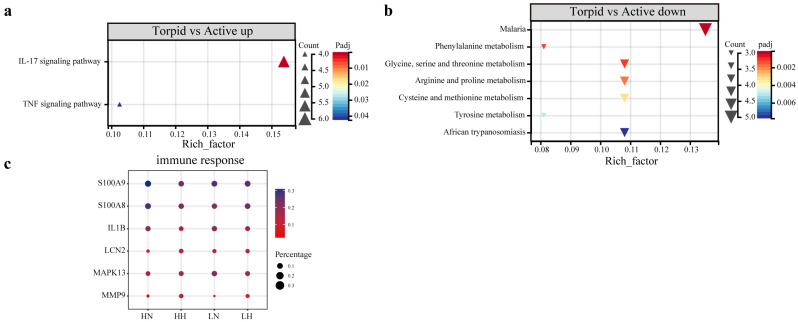
Functional enrichment analysis of DEGs co-regulated in the CE and NE lineages. The KEGG pathway upregulated (**a**) and downregulated (**b**) in torpid vs. active states, and a bubble plot of normalized counts of genes enriched into immune response in each group (**c**).

In the comparative analysis of torpid and active states in the CE lineage (Table S16), four pathways, including platelet activation and the complement and coagulation cascades, are significantly upregulated (Figure 6a), while 50 pathways related to thermogenesis, oxidative phosphorylation, gluconeogenesis, and amino acid metabolism are significantly downregulated (Figure 6b). In contrast, the NE lineage demonstrated significant upregulated in the IL-17 signaling pathway, vitamin B6 metabolism, and tryptophan metabolism (Figure 6c). Additionally, nine KEGG pathways were significantly downregulated, predominantly in categories related to diseases and thermogenic amino acid metabolism, including phenylalanine, glycine, and tyrosine (Table S17, Figure 6d). The co-regulated DEGs are related to immune and metabolic pathways (Table S18), with significant upregulation of the IL-17 and TNF signaling pathways (Figure 7a,c). Meanwhile, seven pathways associated with amino acid metabolism and diseases are significantly downregulated (Figure 7b).

**Figure 8:**
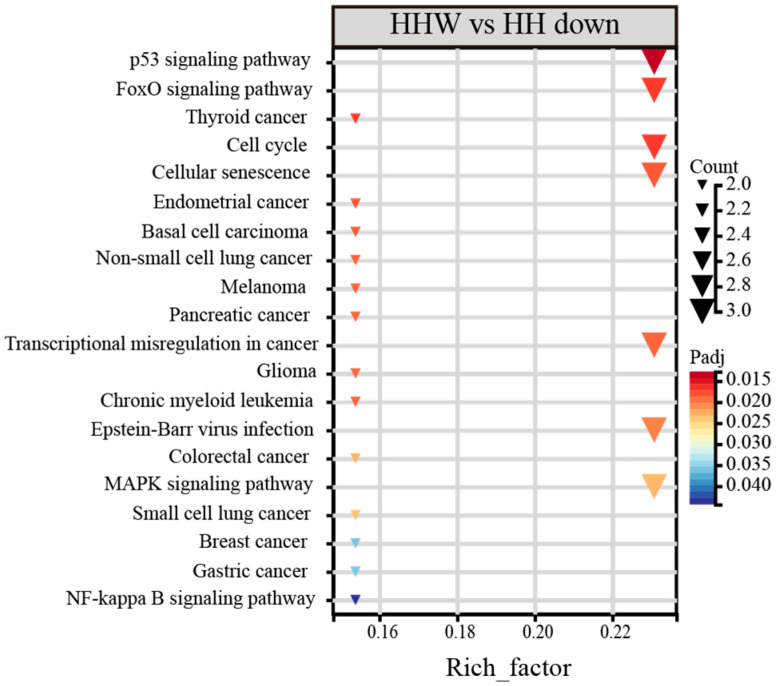
KEGG enrichment pathways of DEGs in arousal vs. torpid states.

In comparing the arousal and torpid states, the CE lineage demonstrated significant enrichment in 22 downregulated KEGG pathways (Table S19). These pathways are predominantly associated with oxidative stress (including MAPK and NF-κB signaling pathways), cell cycle regulation (such as the cell cycle and cellular senescence), and various diseases, among other related pathways (Figure 8). No pathway was significantly enriched in the NE lineage.

The Both_Pos gene module enriched 48 significantly altered metabolic pathways, primarily involving oxidative phosphorylation, thermogenesis, and glycolysis/gluconeogenesis. The Both_Neg gene module enriched 43 significantly altered metabolic pathways, mainly involving protein localisation, organic substance transport, and GTP binding. Neither the HvsN_Only_Pos nor the WvsH_Only_Pos gene modules significantly enriched altered metabolic pathways (Table S20).

## 4. Discussion

### 4.1. Transcriptome Changes Induced by Hibernation Torpor

Hibernating mammals conserve energy during winter by significantly reducing their metabolic rate, which drops to about 4.3% of their normal rate [9], saving around 90% of energy [10]. In hibernating *R. ferrumequinum*, key gluconeogenic enzymes, including *G6PC* and *GOT1*, are downregulated in both lineages, contributing to the inhibition of glucose metabolism [11]. Additionally, the enzyme *AOX1*, which aids in lipid synthesis and tryptophan breakdown [12], is highly expressed, potentially leading to increased levels of kynurenine and NADP [13]. Kynurenine is known to influence bone loss in animals [14].

To combat pathogen threats in humid hibernation environments, *R. ferrumequinum* upregulates certain innate immune pathways and genes, notably IL-17 and TNF signaling pathways. Key genes like *S100A8*/*A9*, which form calprotectin, enhance neutrophil chemotaxis, antibacterial activity, and inflammation [15]. The *BIRC3* gene, part of the inhibitor of apoptosis protein family, regulates cell survival, inflammation, and NF-κB signaling, and is highly expressed in the lymph nodes of hibernating individuals [16]. Different lineages show varied immune gene upregulation; for example, the CE lineage significantly upregulates *HP* and *A2M. HP* provides antibacterial, antioxidant, and pro-angiogenic functions while modulating immune responses [17,18]. *A2M*, a key protease inhibitor in the innate immune system, combats pathogenic proteases by regulating cytokines and lipid mediators [19], boosting anti-inflammatory responses and enhancing innate immunity [20,21]. In the NE lineage, *HPGD*, *HAMP*, and *LEAP2* are significantly upregulated. *HPGD* activates the SOAT3/AKT pathway to reduce inflammatory factors [22]. *HAMP*, crucial for iron metabolism, limits pathogens’ access to iron, providing antimicrobial effects [23]. *LEAP2*, an antimicrobial peptide, directly inhibits bacterial growth [24].

During hibernation torpor, amino acid metabolism is largely suppressed in both lineages due to the downregulation of the pyridoxal phosphate binding pathway, a key coenzyme in amino acid metabolism. This leads to reduced activity in multiple metabolic pathways. The CE lineage primarily downregulates methionine adenosyl transferase 1A (*MAT1A*) and arginosuccinate synthase 1 (*ASS1*), while the NE lineage downregulates genes such as ornithine aminotransferase (*OAT*), and phenylalanine hydroxylase (*PAH*). Both lineages also commonly downregulate tyrosine aminotransferase (*TAT*) and glutamate–oxaloacetate transaminase 1 (*GOT1*). These gene expression changes indicate a significant inhibition of protein synthesis and degradation during hibernation torpor, supporting a hypometabolic state. During torpor, both lineages significantly downregulate the *HBM* gene, weakening core erythrocyte functions like oxygen and heme binding [25]. The lineages regulate iron metabolism differently to tackle hypoxia and maintain iron homeostasis. The CE lineage upregulates ferritin heavy chain 1 (*FTH1*) and hemopexin (*HPX*) to store iron and bind free heme, preventing iron loss and oxidative damage [26,27]. In contrast, the NE lineage upregulates hepcidin (*HAMP*) to reduce plasma iron flux by regulating ferroportin degradation [28,29].

Metabolic regulation during hibernation is not unique to any single species; it is observed in various hibernating animals, such as black bears and ground squirrels [30,31,32]. Upon entering hibernation, the livers of these animals undergo a metabolic shift from carbohydrate metabolism to lipid metabolism, accompanied by a reduction in amino acid catabolism. This metabolic transition represents a common physiological adaptation among hibernating species to cope with cold environments. However, the upregulation of immune functions has been documented only in studies of specific species [5], suggesting that immune response adaptations during hibernation may be species-specific strategies rather than universal features shared by all hibernators.

### 4.2. The Impact of Arousal on the Transcriptome of R. ferrumequinum

The transition from torpor to arousal in *R. ferrumequinum* imposes a series of physiological stresses, including rapid elevation in body temperature, tissue injury due to reperfusion, and oxidative stress. Given that bats must re-enter torpor following a brief homeothermic phase, the number of DEGs between the arousal and torpor states is relatively limited. Despite this limitation, these genes undergo critical adaptive expression changes to address the associated challenges.

Firstly, to meet the high energy demands of the arousal process, a set of genes involved in energy conversion is upregulated. In the CE lineage, the upregulation of *FGF21* promotes fatty acid oxidation, thereby fueling arousal [33]. In the NE lineage, increased expression of genes encoding mitochondrial carrier proteins, such as *SLC25A33* enhances metabolite transport into mitochondria, directly supporting efficient ATP production via oxidative phosphorylation [34]. 

Secondly, a few of innate immune genes (e.g., *HSPH1, PDE4B*) are upregulated to counteract potential infections. Concurrently, to mitigate tissue damage resulting from excessive inflammation, core pro-inflammatory signaling pathways, such as MAPK and NF-κB, are significantly suppressed. This suppression involves the downregulation of key genes: reduced expression of *MAP3K8* directly attenuates the activation of MAPK/NF-κB pathways [35], while downregulation of *GADD45B/G* decreases their ability to activate the p38/JNK pathway via MAP3K4 [36], thereby limiting the drive for inflammation and apoptosis. Different lineages also employ fine-tuning mechanisms through the upregulation of specific genes. For instance, in the CE lineage, *HSPH1* can induce regulatory T cell differentiation to prevent immune overactivation [37]; in the NE lineage, *PDE4B* modulates the pro-inflammatory phenotype of neutrophils and other immune cells by hydrolyzing cAMP [38], thereby aiding in the clearance of latent pathogens.

Finally, the upregulation of certain genes assists in counteracting damage from oxidative stress. For example, *NFE2L2*, a core regulator of the antioxidant response, activates antioxidant enzymes and detoxification genes [39], while *DUSP6* alleviates oxidative stress-induced cell injury by inhibiting the MAPK signaling pathway [40].

### 4.3. Lineage-Specific Regulation of R. ferrumequinum

After the early differentiation of *R. ferrumequinum*, the population expansion north of the Qinling Mountains may have occurred during the Last Glacial Maximum (LGM, 23,000–18,000 years ago), while the population expansion in Henan is estimated to have occurred earliery (20,000–30,000 years ago) [1,3,41]. The Qinling Mountains not only effectively obstruct gene flow but also block the warm and moist air from the southeast, leading to a colder climate on the northern side. The NE population living in higher latitude regions faces more sustained low temperatures and food shortages in winter. Due to the varying pressures of winter, they may achieve hibernation adaptation through different molecular regulatory patterns.

Compared to the CE lineage, the NE lineage of *R. ferrumequinum* exhibited stronger metabolic characteristics and antioxidant capacities during the transition from active states to hibernation. This stable high metabolic profile may underscore the NE lineage’s adaptation to extreme cold while maintaining a more stable hibernation state [42]. The antioxidative defense mediated by superoxide dismutase 1 (*SOD1*), catalase (*CAT*), and peroxiredoxin 6 (*PRDX6*) is crucial for eliminating excess reactive oxygen species (*ROS*) and sustaining redox homeostasis in hibernating mammalian cells, thus protecting the host from oxidative damage [43,44,45,46].

Conversely, the NE lineage appears to downregulate cellular signaling and protein transport regulation, potentially favoring energy conservation. Additionally, during the phase transitions of physiological states, the NE lineage optimizes these transitions by modulating a limited number of genes. In contrast, the CE lineage displays greater flexibility in energy management and environmental adaptability, with a higher number of DEGs, including significant downregulation of the *PCK1* and *APOE* genes, which reduces glucose metabolism and promotes dormancy during hibernation. *PCK1* is critical for energy metabolism and gluconeogenesis, while several thermogenic amino acid pathways are also inhibited [47,48], allowing for a more dynamic response to changing conditions.

## 5. Conclusions

Our research provided important insights into the molecular adaptations related to hibernation in two major lineages of *R. ferrumequinum*. Liver transcriptome analysis shows that during torpor, both lineages shift their metabolism from carbohydrates to lipids, reduce amino acid metabolism, and upregulate immune-related genes to enhance pathogen defense. However, when arousing from torpor, only a few immune and metabolic genes are activated, helping them recover quickly. The limited overlap of DEGs between the lineages suggests unique evolutionary paths, likely reflecting specific environmental adaptations. Unexpectedly, the NE lineage, which inhabits colder regions, showed significantly fewer DEGs across all stages compared to the CE lineage. This may be due to the NE lineage’s adaptation to long-term energy constraints and cold temperatures, while the CE lineage may need to regulate more genes to cope with environmental fluctuations and physiological challenges.

However, this study is primarily based on transcriptomic data from liver tissue, which may limit the interpretation of our findings. Future research should incorporate proteomics or metabolomics to validate our results. Additionally, it is essential to investigate other tissues, such as the brain, gut, skeletal muscle, and adipose tissue, as they may play significant roles in lineage-specific regulation. Moreover, given the substantial impact of environmental factors like humidity and temperature on animal physiology, future research could integrate single-cell and spatial transcriptomics with measurements of microclimate variables and functional characteristics. This comprehensive approach will enhance our understanding of the combined effects of genetics and environment on lineage-specific hibernation strategies.

## Data Availability

The original data presented in the study are openly available in the National Center for Biotechnological Information’s Short Read Archive under BioProject ID CRA034567 at [https://ngdc.cncb.ac.cn/gsa/] (accessed on 2 March 2026).

## Supplementary Materials

The following supporting information can be downloaded at https://www.mdpi.com/article/10.3390/biology15050425/s1, Table S1 Individual Information. Table S2 Sample transcriptome sequencing information. Table S3 Information of the upregulated and downregulated DEGs for HH (CE lineage). Table S4 Information of the upregulated and downregulated DEGs for LH (NE lineage). Table S5 Information of the upregulated and downregulated DEGs for HHW (CE lineage). Table S6 Information of the upregulated and downregulated DEGs for LHW (NE lineage). Table S7 Top 10 in the network by maximum cluster centrality (MCC) algorithm. Table S8 Co-regulated upregulated and downregulated DEGs in the hibernation torpor groups. Table S9 The time model results of DEAeq2 reveal genes that exhibit lineage-specific regulation across different physiological stages. Table S10 GO terms enriched in CE lineage (torpor vs. active). Table S11 GO terms enriched in NE lineage (torpor vs. active). Table S12 Co-regulated GO Terms for Two Lineages (torpor vs. active). Table S13 GO terms enriched in CE lineage (arousal vs. torpor). Table S14 GO terms enriched in NE lineage (arousal vs. torpor). Table S15 GO terms significantly enriched in different regulatory modes. Table S16 KEGG pathways enriched in CE lineage (torpor vs. active). Table S17 KEGG pathways enriched in NE lineage (torpor vs. active). Table S18 Co-regulated KEGG pathways for Two Lineages (torpor vs. active). Table S19 KEGG pathways enriched in CE lineage (arousal vs. torpor). Table S20 KEGG pathways significantly enriched in different regulatory modes. Figure S1 Cluster heatmap of DEGs from active to torpor stage (CE lineage). Figure S2 Cluster heatmap of DEGs from active to torpor stage (NE lineage). Figure S3 Cluster heatmap of DEGs from torpor to arousal stage (CE lineage). Figure S4 Cluster heatmap of DEGs from torpor to arousal stage (NE lineage). Figure S5 Cluster heatmap of DEGs co-regulated by both lineages from active to torpor stage. Figure S6 Cluster heatmap of genes exhibiting lineage-specific regulation at different physiological stages. Figure S7 Expression trends of TOP9 genes in the four modes of lineage-specific regulation.
