# Supplementary material for: Hepatic Transcriptome Variations Among Different Evolutionary Lineages of Rhinolophus ferrumequinum During Hibernation
Source: Biology (Basel). 2026 Mar 5;15(5):425. doi: 10.3390/biology15050425 (PMC12984447; doi:10.3390/biology15050425)
Supplement: Supplementary file 1 [file biology-15-00425-s001.zip › Supplemental information.pdf]

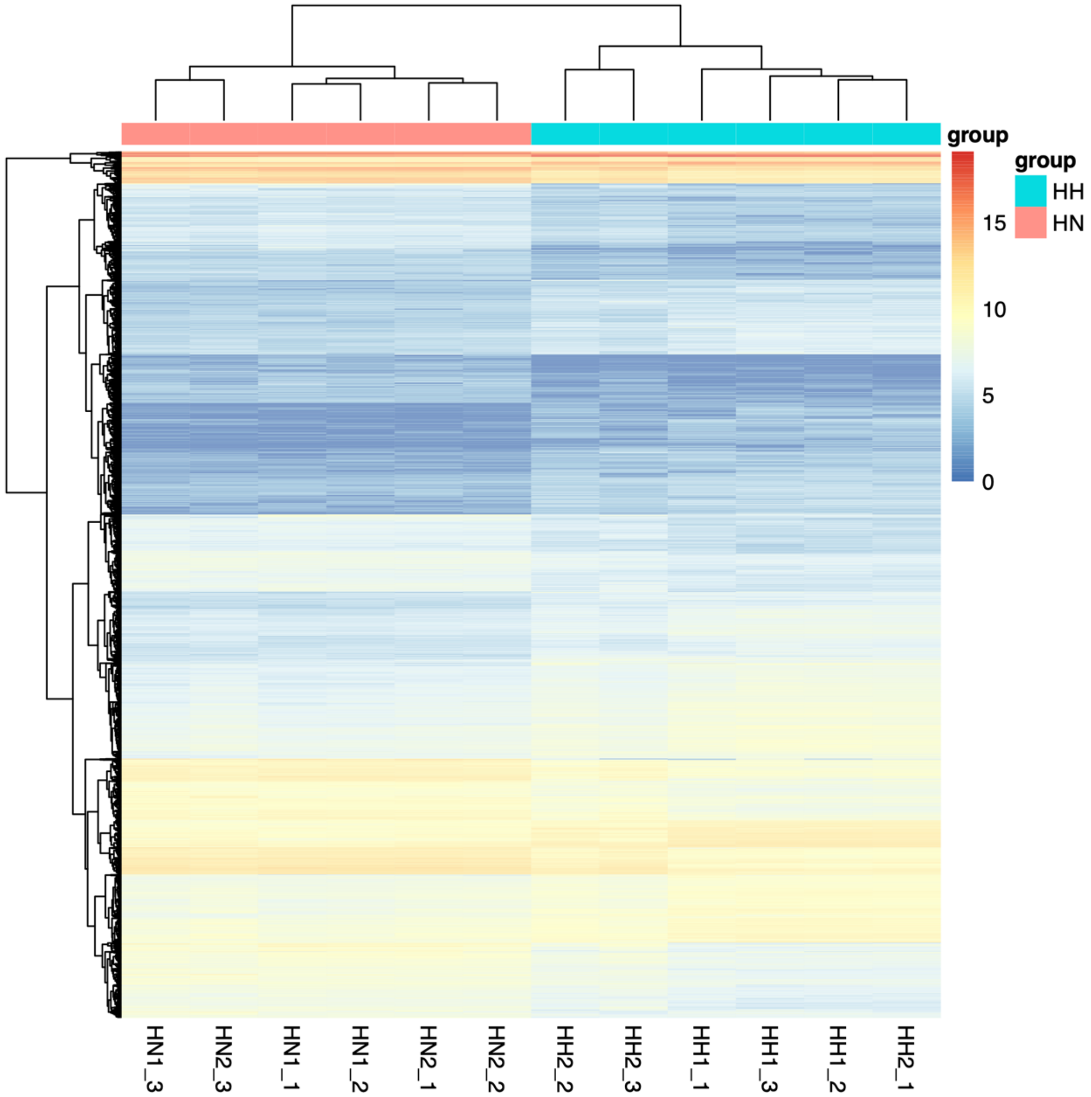

Supplementary Figure S1 Cluster heatmap of DEGs from active to torpor stage (CE lineage)

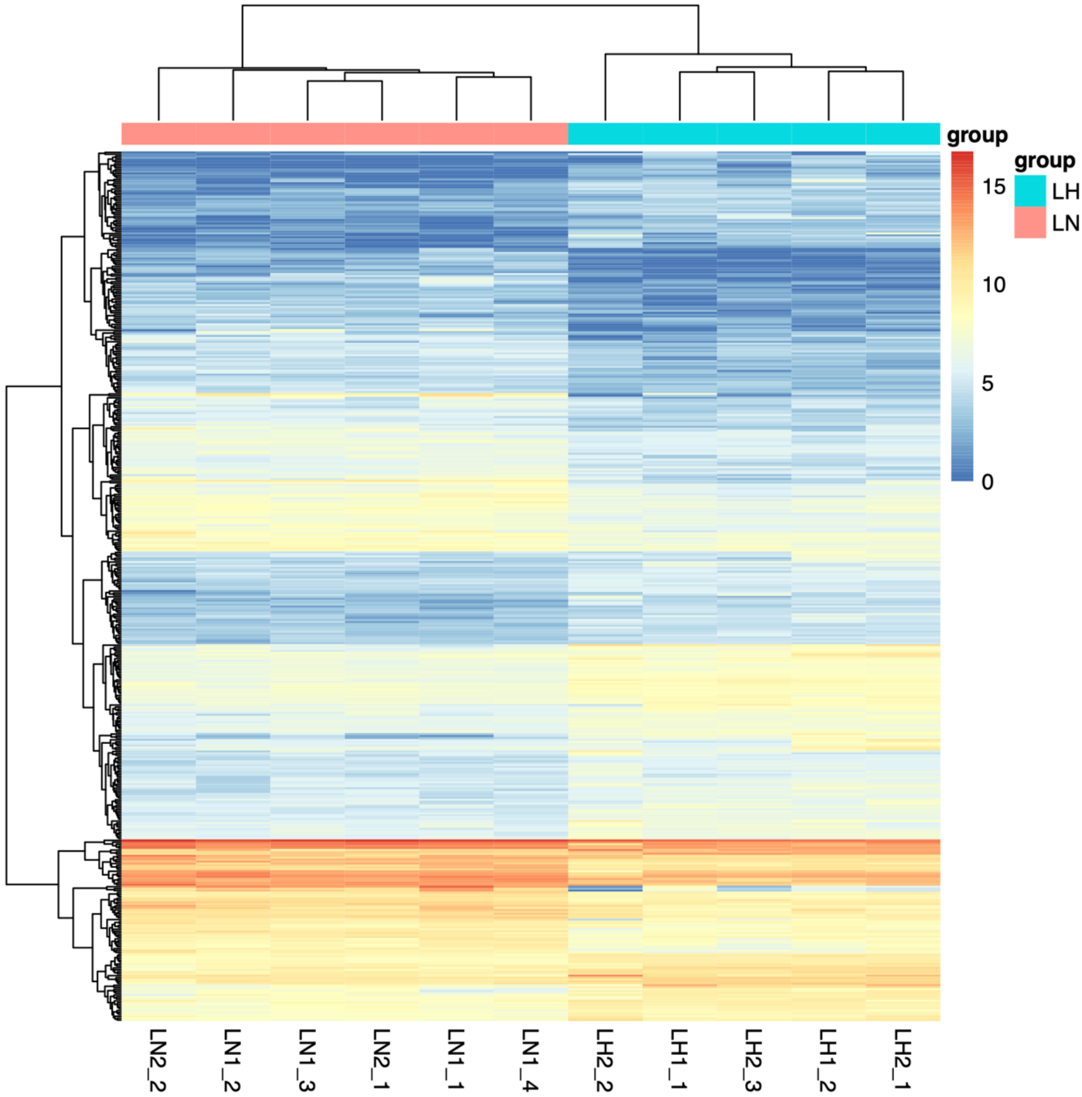

Supplementary Figure S2 Cluster heatmap of DEGs from active to torpor stage (NE lineage)

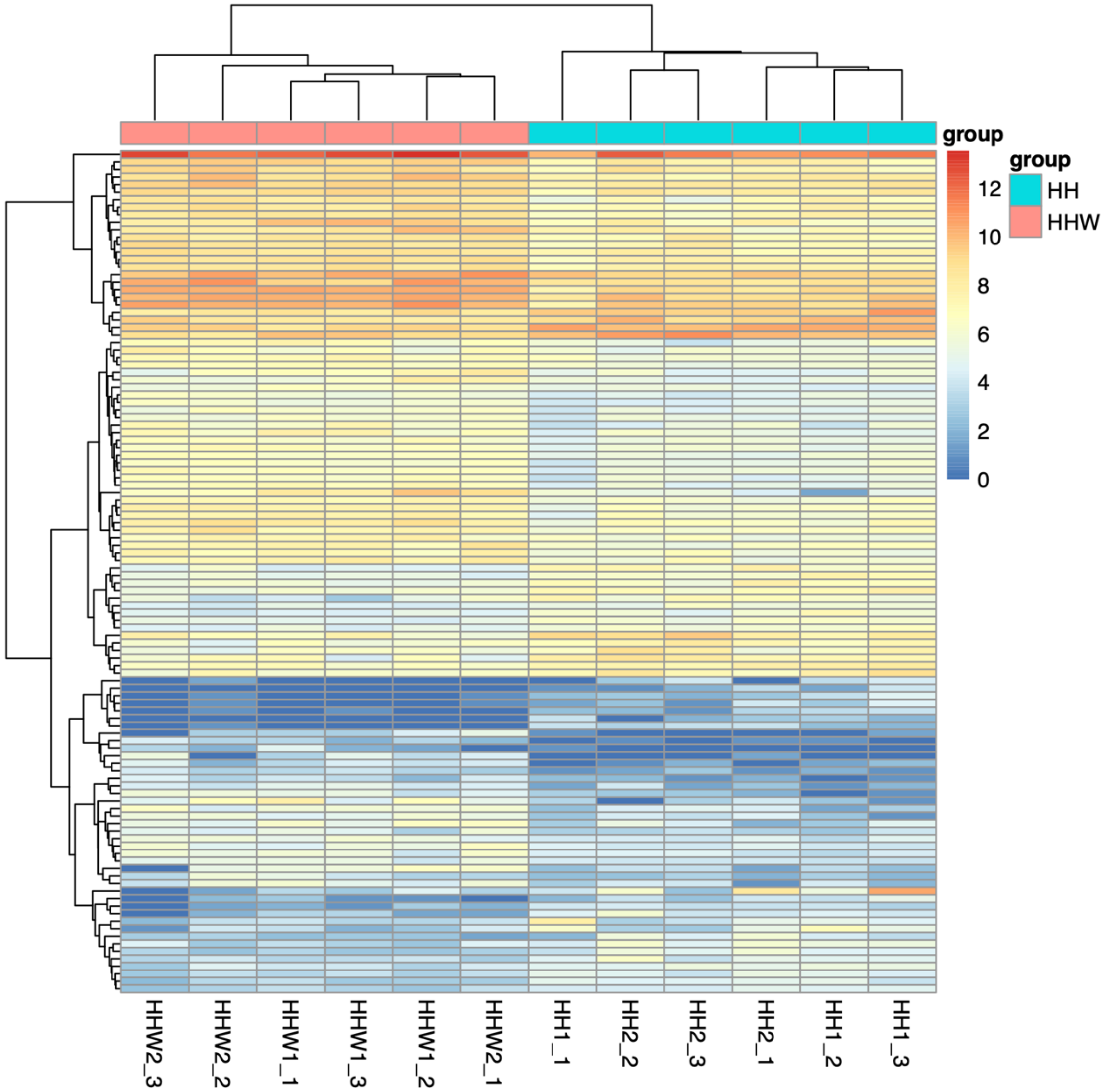

Supplementary Figure S3 Cluster heatmap of DEGs from torpor to arousal stage (CE lineage)

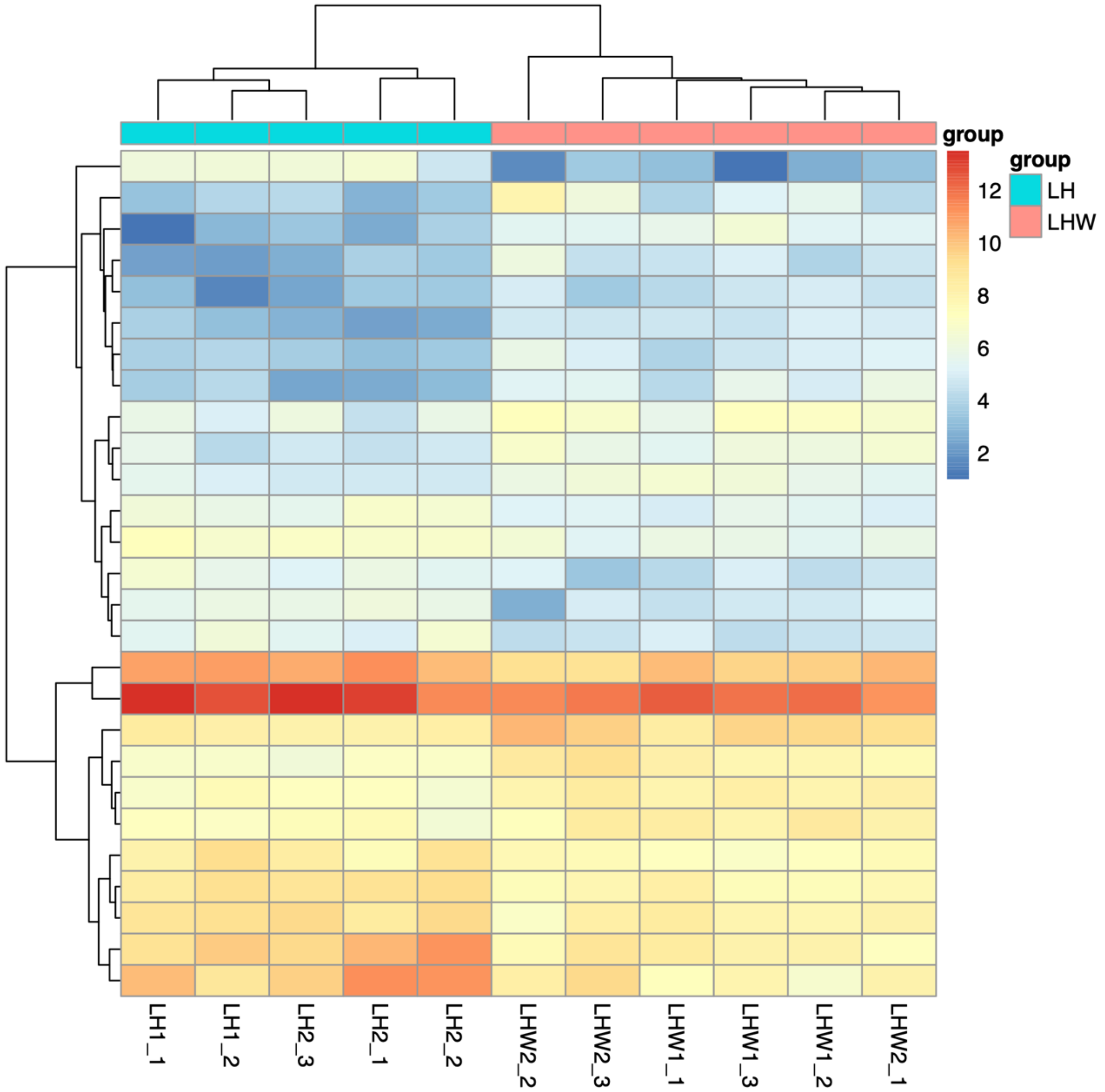

Supplementary Figure S4 Cluster heatmap of DEGs from torpor to arousal stage (NE lineage)

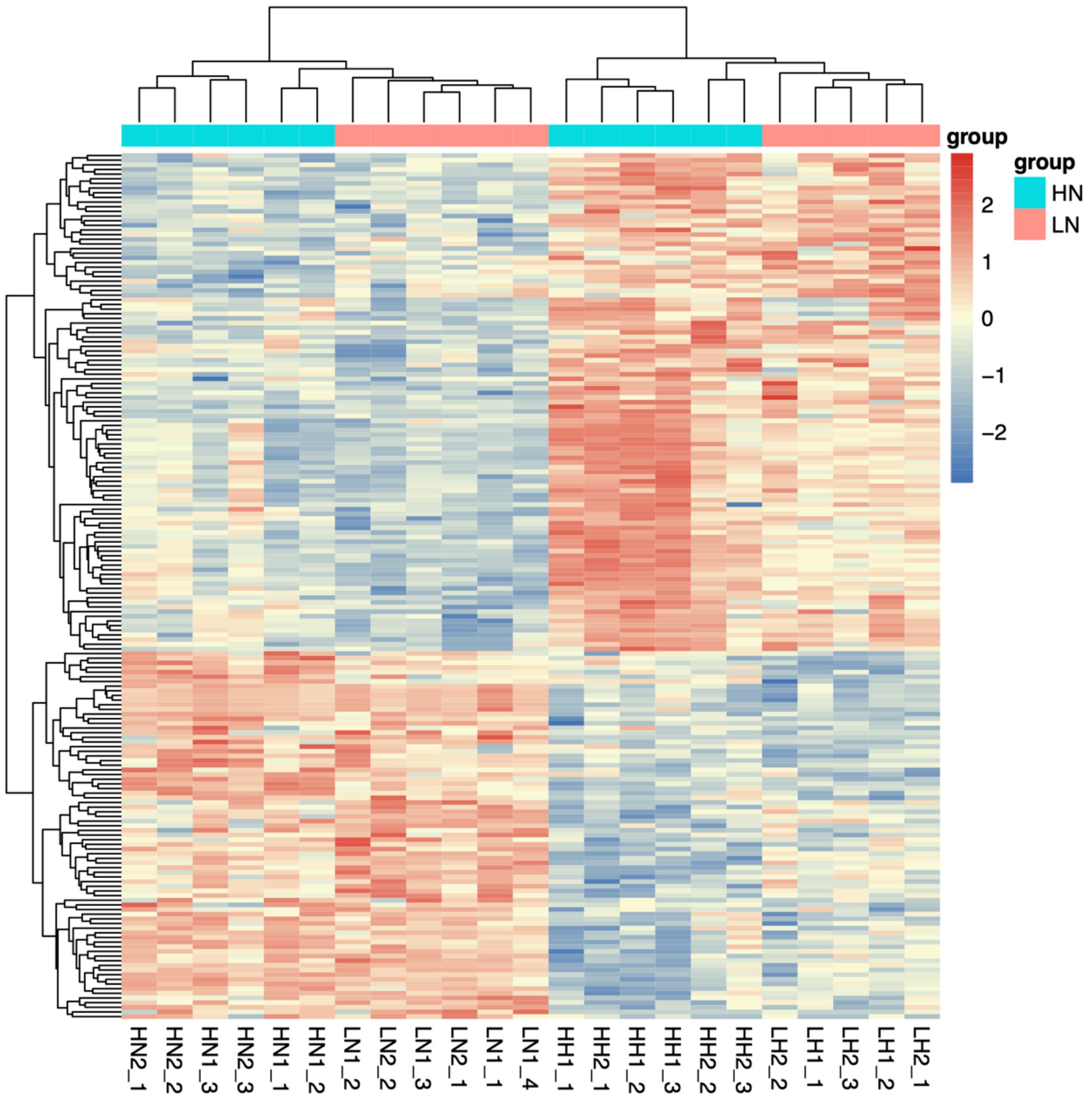

Supplementary Figure S5 Cluster heatmap of DEGs co-regulated by both lineages from active to torpor stage

Supplementary Table S7 Top 10 in the network by maximum cluster centrality (MCC) algorithm

Rank	HH vs HN	LH vs LN	HHW vs HH
1	*SMC2*	*ISG15*	*RND3*
2	*SMC4*	*OASL*	*SGK1*
3	*TOP2A*	*XAF1*	*TRIB1*
3	*MCM3*	*MX2*	*CCDC39*
5	*AURKB*	*EIF2AK2*	*ASCL1*
6	*NCAPH*	*IFIT1*	*NFE2L2*
7	*PLK4*	*IFI44L*	*GADD45B*

8	*CCNB2*	*SAMD9*	*RASD2*
9	*ESPL1*	*HERC6*	*KCNS3*
10	*TTK*	*ATM*	*CD79B*

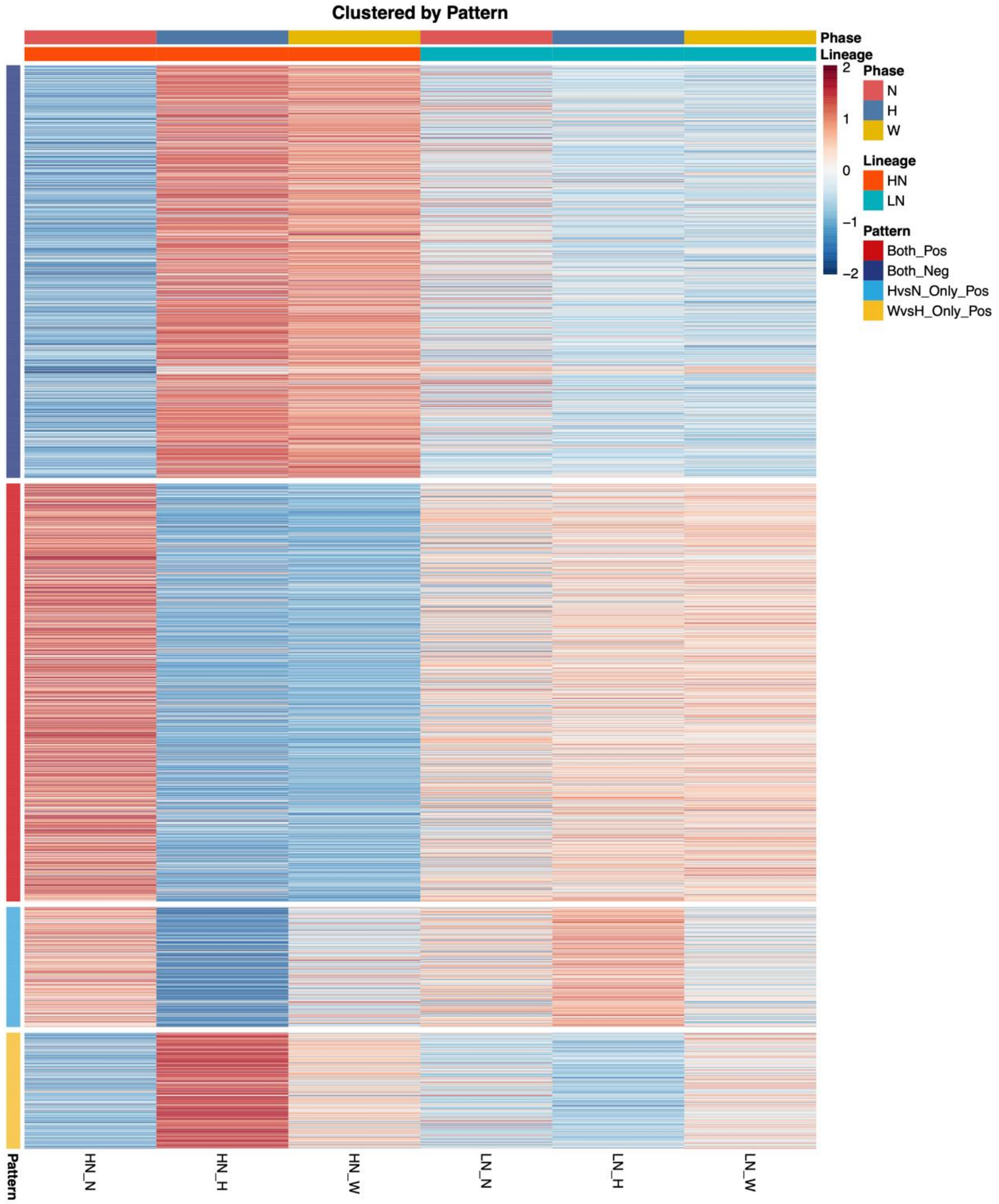

Supplementary Figure S6 Cluster heatmap of genes exhibiting lineage-specific regulation at different physiological stages. N represents active, H represents torpid, and W represents arousal. The CE lineage (HN) is orange, while the NE lineage (LN) is blue.

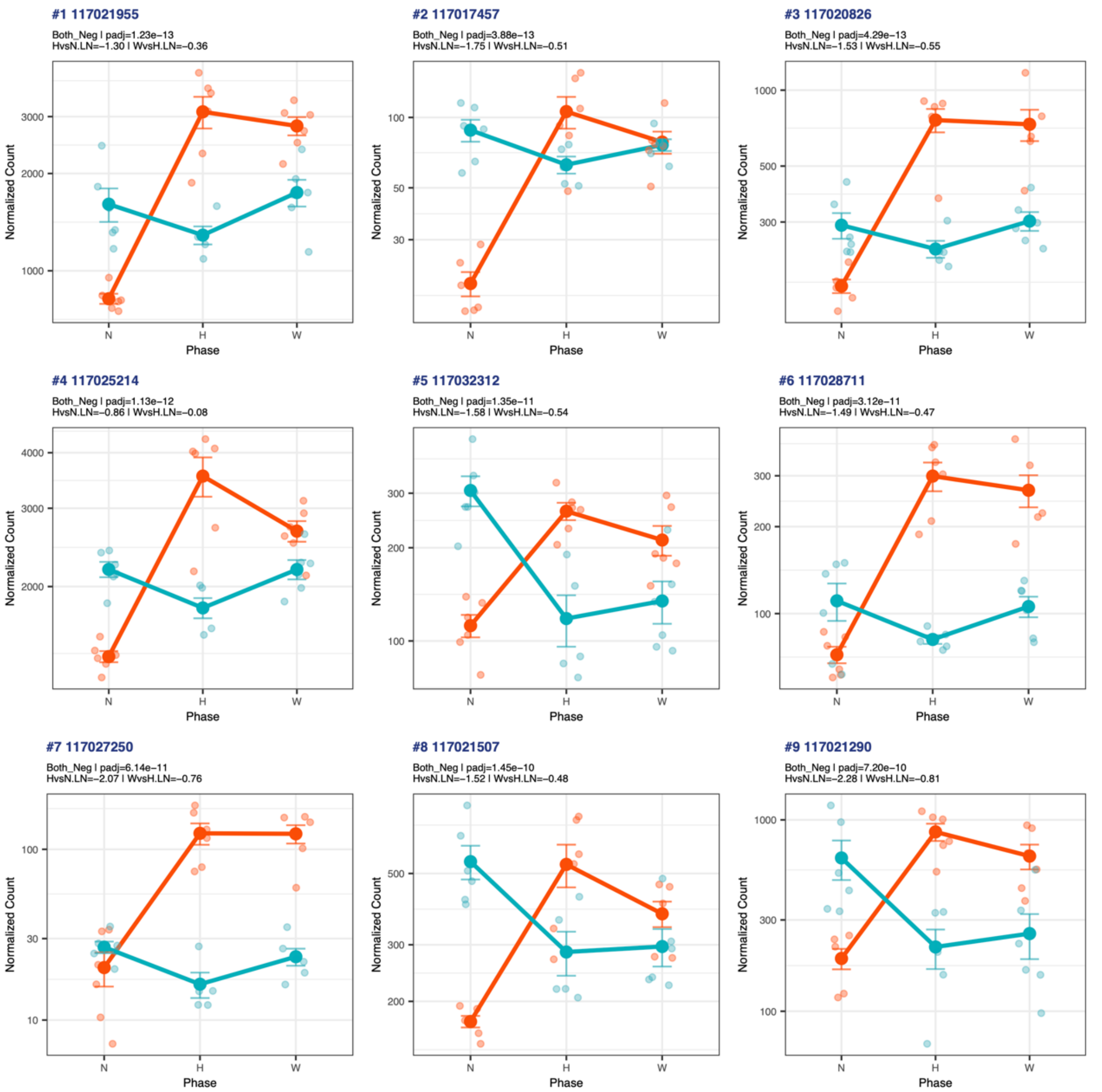

Supplementary Figure S7 Expression trends of TOP9 genes in the four modes of lineage-specific regulation. The CE lineage (HN) is orange, while the NE lineage (LN) is blue. Gene name is NCBI's Gene ID.
